# Severe Hyponatremia Caused by the Syndrome of Inappropriate Antidiuresis Due to Urinary Retention

**DOI:** 10.7759/cureus.73589

**Published:** 2024-11-13

**Authors:** Takuya Kumagai, Hitomi Miyashita, Uran Shimada, Tsuyoshi Ono

**Affiliations:** 1 Department of Hematology, Nephrology and Rheumatology, Akita University Graduate School of Medicine, Akita, JPN; 2 Department of Internal Medicine, Municipal Omori Hospital, Yokote, JPN; 3 Clinical Training Center, Hiraka General Hospital, Yokote, JPN

**Keywords:** differential diagnosis of hyponatremia, siad, symptomatic hyponatremia, syndrome of inappropriate adh secretion, urinary retention (ur)

## Abstract

We present a case of a 90-year-old female patient with severe hyponatremia due to syndrome of inappropriate antidiuresis (SIAD) induced by urinary retention. The patient had a history of neurogenic bladder and developed fatigue that progressively worsened over 10 days prior to admission. Initial treatment with hypertonic saline and bladder catheterization led to improvements in her serum sodium levels and symptoms. Based on her clinical course and laboratory findings, urinary retention was determined to be the underlying cause of SIAD. This case highlights that, although rare, urinary retention should be considered a potential trigger for SIAD in patients with unexplained hyponatremia. After relieving urinary retention, suppression of antidiuretic hormone secretion can occur, sometimes leading to rapid correction of serum sodium levels. Therefore, careful monitoring of sodium levels is required to prevent complications such as osmotic demyelination syndrome.

## Introduction

Syndrome of inappropriate antidiuresis (SIAD) is a common cause of hyponatremia and is typically associated with conditions such as malignancies, medications, and pulmonary diseases [1]. Although urinary retention is a rare and often underrecognized cause of SIAD, several case reports have documented this association. While the precise mechanism linking urinary retention and antidiuretic hormone (ADH) secretion remains unclear, two pathways have been proposed in the pathophysiology of SIAD from urinary retention: ADH secretion induced by pain from bladder distention and sympathetic activation from bladder wall stretch, both of which lead to ADH release [2]. It is essential to recognize urinary retention as a potential cause of SIAD, as the rapid cessation of ADH secretion following relief of retention can result in swift sodium correction, posing a risk for osmotic demyelination syndrome (ODS).

## Case presentation

A 90-year-old female patient presented with difficulty urinating involving a sensation of the urge to urinate but only being able to produce a small amount, with a persistent feeling of residual urine. She was diagnosed with neurogenic bladder by her local urologist two weeks prior. She was prescribed an alpha-blocker, but her symptoms showed minimal improvement. Ten days before admission, she developed an unsteady gait and became unable to take her regular medications. Three days prior to admission, she exhibited lethargy and disorientation, accompanied by headache and nausea, prompting a visit to her primary care physician. Blood tests revealed severe hyponatremia, with a serum sodium level of 111 mEq/L, and she was referred to our hospital. Her medical history included angina pectoris and hypertension, for which she had been prescribed amlodipine 5 mg/day, valsartan 80 mg/day, hydrochlorothiazide 6.25 mg/day, and all of these medications had been continued for more than five years. However, she had not taken these medications for the past 10 days.

The patient was an elderly woman, 143 cm tall, weighing 45.3 kg, with a BMI of 22.1 kg/m^2_. _^On admission, her vital signs were as follows: Japan Coma Scale (JCS) I-2, Glasgow Coma Scale (GCS) score was 14, with E4V4M6, heart rate 90 bpm, body temperature 36.6°C, blood pressure 144/79 mmHg, and oxygen saturation 99% on ambient air. She displayed no signs of oral or tongue dryness. Heart and lung sounds were normal, a soft mass was palpable in the lower abdomen, and there was no peripheral edema. Her physique was typical for her age, with no signs of cachexia.

Laboratory results are summarized in Table 1. 

**Table 1:** Initial blood and urine test results. ACTH: adrenocorticotropic Hormone, Alb: albumin, BS: blood sugar, BUN: blood urea nitrogen,  CRP: C-reactive protein, FT3: free T3, FT4: free T4, Hb: hemoglobin, Hct: hematocrit, PLT: platelet,  RBC: red blood cell, TP: total protein, TSH: thyroid stimulating hormone, UA: uric acid, WBC: white blood cell

Test	Result	Reference Values
TP	7.3 g/dL	6.4-8.3 g/dL
Alb	4.5 g/dL	3.5-5.0 g/dL
Cr	0.64 mg/dL	0.6-1.3 mg/dL
BUN	17 mg/dL	7-20 mg/dL
UA	4.0 mg/dL	2.5-7.5 mg/dL
Na	111 mEq/L	135-145 mEq/L
K	3.8 mEq/L	3.5-5.0 mEq/L
Cl	75 mEq/L	98-107 mEq/L
BS	116 mg/dL	<140 mg/dL
Osmolality	229 mOsm/L	275-295 mOsm/L
WBC	10420 /μL	4000-10000 /μL
RBC	372 ×10⁴/μL	380-520 ×10⁴/μL
Hb	11.8 g/dL	12-16 g/dL
Hct	30.1 %	35.1-44.4 %
PLT	26.9 ×10⁴/μL	150-450 ×10⁴/μL
ACTH	45.8 pg/mL	7.2-63.3 pg/mL
Cortisol	16.3 μg/dL	5-25 μg/dL
TSH	0.516 μIU/mL	0.4-4.0 μIU/mL
FT3	2.38 ng/mL	2.0-4.4 ng/mL
FT4	1.88 pg/mL	0.8-1.8 pg/mL
Renin	3.5 ng/mL/hr	0.5-3.3 ng/mL/hr
Aldosterone	7.3 pg/mL	4-31 pg/mL
Urine osmolality	240 mOsm/L	50-1200 mOsm/L
Urine sodium	48 mEq/L	40-220 mEq/L

Severe hyponatremia was confirmed with a serum sodium level of 111 mEq/L. Plasma osmolality was 229 mOsm/L (reference range: 280-290 mOsm/L), indicating hypotonic hyponatremia. Urine osmolality was elevated at 240 mEq/L. Mild hyperthyroidism was noted, but adrenal function was within normal limits.

**Figure 1:**
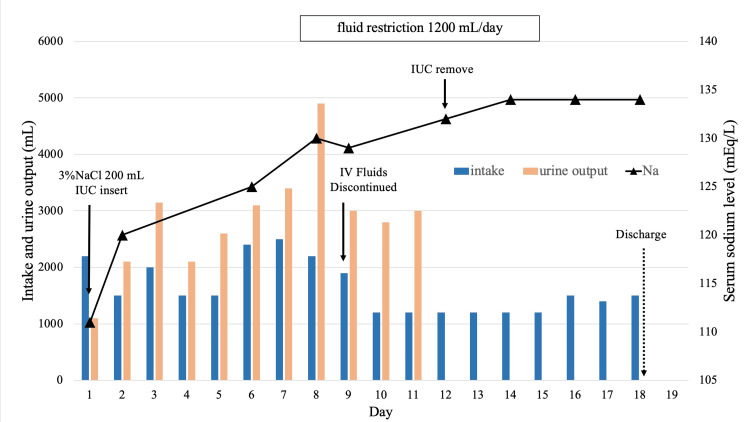
Trends in the serum sodium level and fluid balance during treatment. IUC: indwelling urinary catheter, IV: Intravenous injection Intake: Total fluid intake, including oral intake and intravenous fluids, excluding dietary fluids.

Chest X-ray and electrocardiogram showed no significant abnormalities. CT imaging revealed a markedly distended bladder due to urinary retention. An indwelling urinary catheter was inserted, draining approximately 300 mL of urine immediately. For symptomatic severe hyponatremia, a 100 mL bolus of 3% NaCl was administered. After one hour, her serum sodium level had risen slightly to 112 mEq/L, with minimal improvement in symptoms due to hyponatremia, such as headache and nausea. A second 100 mL bolus of 3% NaCl was therefore administered. Two hours later, the serum sodium level had increased to 116 mEq/L, accompanied by symptom improvement. By the following day, her serum sodium level had risen to 120 mEq/L. Despite the rapid correction, no further treatment was given, as her symptoms improved and she could regulate fluid intake according to thirst. By day 5, her serum sodium level had reached 125 mEq/L, and by day 7, it had further risen to 130 mEq/L. However, on day 8, her sodium level dropped slightly to 129 mEq/L, leading to the initiation of fluid restriction at 1200 mL per day. Consequently, her serum sodium level gradually increased, reaching 134 mEq/L by day 13. The fluid restriction was lifted on day 15, and no subsequent decline in serum sodium levels was observed by day 17 (Figure 1). 

The patient’s volume status was euvolemic, and based on her clinical course and laboratory findings, with observed improvement in serum sodium following water restriction, SIAD was identified as the most probable diagnosis. Thiazide-induced hyponatremia was initially considered; however, this was deemed unlikely as the patient had not taken her medications for the 10 days preceding admission. No other causes of hyponatremia were identified, and with the improvement in sodium levels after resolving urinary retention, urinary retention was concluded to be the underlying cause of her SIAD. No recurrence of hyponatremia was observed after discharge.

Regarding the treatment of urinary retention, the alpha-blocker dosage was adjusted after admission, and the indwelling bladder catheter was removed on day 11 of hospitalization. The patient subsequently experienced no recurrence of urinary retention and was discharged on day 18. Follow-up care will continue on an outpatient basis.

## Discussion

We present a case of SIAD triggered by urinary retention. Although malignancies, medications, and pulmonary diseases are widely recognized causes of SIAD, urinary retention is not currently addressed in clinical guidelines [1]. To the best of our knowledge, 20 cases have been previously reported, which are shown in Table 2 [2-13]. 

**Table 2:** Case reports on hyponatremia secondary to urinary retention. BPH: benign prostatic hyperplasia, F: female, ICD: indwelling urinary catheter, M: male, ND: no data, Ref: reference number, sNa: serum sodium level

References	Age/Sex	sNa level	Cause of urinary retention	Treatment otherwise IUC	Time to sNa normalization	Year
Moskowitz [3]	42/F	115	Functional obstructive uropathy	0.9% saline	7 hours	1992
Galperin et al. [4]	78/F	107	ND	ND	ND	2007
Galperin et al. [4]	83/F	118	ND	ND	ND	2007
Galperin et al. [4]	78/M	119	ND	ND	ND	2007
Galperin et al. [4]	92/M	121	ND	ND	ND	2007
Galperin et al. [4]	91/M	127	ND	ND	ND	2007
Galperin et al. [4]	90/F	129	ND	ND	ND	2007
Erza and Alessi [5]	80/M	120	BPH	Demeclocycline	10 days	2009
Lax, Kinderknecht, and Titus [2]	83/M	111	BPH	3% saline	15 hours	2014
Mahajan and Simon [6]	76/M	120	BPH	None	2 days	2014
Irani et al. [7]	31/F	121	Impacted gravid uterus	0.45% saline	4 hours	2016
Parikh et al. [8]	63/M	105	BPH	1.5% saline	5 days	2017
Parikh et al. [8]	40/F	120	Paraplegia after laminectomy	1.5% saline	12 hours	2017
Hogan et al. [9]	83/F	115	Atonic bladder	None	9 days	2019
Hilton et al. [10]	60s/F	98	drug induced or urinary tract infection	0.9% saline	10 days	2020
Saleh et al. [11]	52/M	103	Unknown	3% saline	ND	2020
van der Bilt and Alsma[12]	32/M	112	Anticholinergic syndrome	8.4% sodium bicarbonate, desmopressin	2 days	2023
van der Bilt and Alsma [12]	82/M	113	Urological anatomical abnormality	3% saline, 5% glucose infusion	ND	2023
van der Bilt and Alsma [12]	56/M	108	Possible stress/ alcohol abuse	Desmopressin	ND	2023
Jannat, Hussain and Ahmad [13]	50/F	118	Intramural uterine fibroid	0.9% saline, 5% glucose infusion	10 hours	2024
Our case	90/F	111	Neurogenic bladder	3% saline	13 days	2024

The median patient age was 78 years (range 31-92), with 57.9% being male. The most frequent cause of urinary retention was benign prostatic hyperplasia (BPH), and the median serum sodium level was 116.5 mEq/L (range 98-129). In each case, SIAD improved following bladder catheterization. Severe hyponatremia, with serum sodium levels below 120 mEq/L, was observed in 12 of the 20 cases.

While the precise mechanism linking urinary retention and ADH secretion remains unclear, two possible explanations have been proposed for SIAD triggered by urinary retention. The first mechanism proposes that pain stimuli from bladder distention directly induce ADH secretion, while the second suggests that bladder wall stretching itself triggers sympathetic nervous system activation, which then stimulates ADH secretion [5]. Furthermore, once urinary retention is resolved, ADH suppression leads to rapid free water excretion and a consequent rise in serum sodium levels. In previous reports, seven of the 20 cases showed an increase in serum sodium of more than 10 mEq/L per day. Therefore, measures such as administering desmopressin or a 5% dextrose solution have been employed to control the rate of sodium correction and mitigate the risk of ODS. The rapid onset of water diuresis following urinary retention relief likely stems from the swift decrease in plasma ADH levels, given that ADH has a short half-life of approximately 16 to 20 minutes [14].

In our case, despite severe hyponatremia with a serum sodium level of 111 mEq/L, the patient exhibited only mild symptoms, suggesting a chronic course of hyponatremia, likely due to chronic urinary dysfunction prior to admission, which may have resulted in mild but sustained ADH secretion. This ongoing ADH stimulation might explain why this case did not experience the rapid sodium correction seen in previous reports after relieving urinary retention. Additionally, while risk factors for ODS, such as hypokalemia, alcoholism, and liver disease, generally require a slow correction rate of approximately 6 mEq/L per day [15], our patient lacked such risk factors. Although the administration of 3% hypertonic saline was required to improve symptoms of cerebral edema, we determined that overly rapid correction of serum sodium levels was unlikely, as the patient was able to appropriately regulate her water intake in response to thirst following symptom improvement.

Although current domestic and international guidelines do not identify urinary retention as a potential cause of SIAD, several case reports indicate that it may contribute to the syndrome [12]. Undiagnosed cases may be common, and clinicians should recognize urinary retention as a possible contributor to SIAD to reduce the risk of ODS due to rapid sodium correction.

Recent reports indicate that while rapid correction of severe hyponatremia is relatively common, the occurrence of ODS remains rare [16]. However, cases of ODS have been associated with significantly lower initial serum sodium levels, suggesting that severe hyponatremia itself may be a risk factor for ODS [16]. Previous reports also show that hyponatremia due to urinary retention often presents with severe cases. Therefore, careful monitoring and attention to correction rates are essential to ensure patient safety.

## Conclusions

This case underscores urinary retention as a potential but often underrecognized trigger for SIAD, especially in patients with unexplained hyponatremia. Although rare, urinary retention-induced SIAD should be considered in the differential diagnosis due to the associated risk of severe hyponatremia and the possibility of rapid serum sodium correction following bladder decompression. The clinical course in this case suggests that longstanding urinary dysfunction may lead to chronic, mild ADH stimulation, which might reduce the likelihood of an immediate, significant sodium correction response often seen after urinary retention relief.

The risk of ODS associated with the rapid correction of severe hyponatremia, particularly in cases with significantly low initial serum sodium levels, highlights the importance of careful management and monitoring. Clinicians should be vigilant about the potential for abrupt ADH suppression and subsequent free water diuresis after resolving urinary retention, as this may require controlled serum sodium correction to prevent complications. Recognizing urinary retention as a possible contributor to SIAD can enhance clinical outcomes by enabling proactive management strategies that prioritize patient safety and mitigate the risks of rapid serum sodium fluctuations.
